# First report of *Klebsiella pneumoniae* co-producing OXA-181, CTX-M-55, and MCR-8 isolated from the patient with bacteremia

**DOI:** 10.3389/fmicb.2022.1020500

**Published:** 2022-10-14

**Authors:** Haoyu Ge, Jie Qiao, Hao Xu, Ruishan Liu, Ruyan Chen, Chenyu Li, Xinjun Hu, Jiawei Zhou, Xiaobing Guo, Beiwen Zheng

**Affiliations:** ^1^Collaborative Innovation Center for Diagnosis and Treatment of Infectious Diseases, State Key Laboratory for Diagnosis and Treatment of Infectious Diseases, The First Affiliated Hospital, College of Medicine, Zhejiang University, Hangzhou, China; ^2^Department of Laboratory Medicine, The First Affiliated Hospital of Zhengzhou University, Zhengzhou, China; ^3^Department of Infectious Diseases, The First Affiliated Hospital, College of Clinical Medicine, Henan University of Science and Technology, Luoyang, China; ^4^Department of Structure and Morphology, Jinan Microecological Biomedicine Shandong Laboratory, Jinan, Shandong, China; ^5^Research Units of Infectious Diseases and Microecology, Chinese Academy of Medical Sciences, Beijing, China

**Keywords:** *Klebsiella pneumoniae*, OXA-181, CTX-M-55, MCR-8, bacteremia

## Abstract

The worldwide spread of carbapenem-resistant Enterobacteriaceae (CRE) has led to a major challenge to human health. In this case, colistin is often used to treat the infection caused by CRE. However, the coexistence of genes conferring resistance to carbapenem and colistin is of great concern. In this work, we reported the coexistence of *bla*_OXA-181_, *bla*_CTX-M-55_, and *mcr-8* in an ST273 *Klebsiella pneumoniae* isolate for the first time. The species identification was performed using MALDI-TOF MS, and the presence of various antimicrobial resistance genes (ARGs) and virulence genes were detected by PCR and whole-genome sequencing. Antimicrobial susceptibility testing showed that *K. pneumoniae* 5589 was resistant to aztreonam, imipenem, meropenem, ceftriaxone, cefotaxime, ceftazidime, levofloxacin, ciprofloxacin, gentamicin, piperacillin-tazobactam, cefepime, and polymyxin B, but sensitive to amikacin. S1-pulsed-field gel electrophoresis (PFGE) and Southern blotting revealed the *mcr-8* gene was carried on a ~ 138 kb plasmid with a conserved structure (IS*903B*-*ymoA*-*inhA*-*mcr-8*-*copR*-*baeS*-*dgkA*-*ampC*). In addition, *bla*_OXA-181_ was found on another ~51 kb plasmid with a composite transposon flanked by insertion sequence IS*26*. The *in vitro* conjugation experiments and plasmid sequence probe indicated that the plasmid p5589-OXA-181 and the p5589-mcr-8 were conjugative, which may contribute to the propagation of ARGs. Relevant detection and investigation measures should be taken to control the prevalence of pathogens coharboring *bla*_OXA-181_, *bla*_CTX-M-55_ and *mcr-8*.

## Introduction

As one of the significant challenges to global public health, bacterial resistance has attracted much attention in clinical treatment (Xiao et al., 2016; Lai et al., 2021). Especially the infection caused by carbapenem-resistant Enterobacteriaceae (CRE) puts pressure on the health care system in China (Zheng et al., 2018, 2019a; Tompkins and van Duin, 2021).

OXA-48, one of the most common carbapenemases, was first reported in a *K. pneumoniae* isolated from a patient in Turkey (Mairi et al., 2018). OXA-48, unlike the other major carbapenemases, is an ambler class D enzyme that shows low activity against carbapenems and spares extended-spectrum cephalosporins (Stewart et al., 2018). Therefore, it is challenging to detect *bla*_OXA-48_-habouring bacteria clinically. Till now, OXA-48 has more than 10 variants, and OXA-181 is currently the second most common global derivative, which differs from OXA-48 by four amino acid substitutions (Messaoudi et al., 2021). Unlike the prevalence of KPC, NDM and IMP, OXA-181 mainly occurs in India, Europe and the South-East Mediterranean region (Nigg et al., 2019; Shanthini et al., 2019). The emergence of *bla*_OXA-181_ in China has aroused concern extensively.

Extended-spectrum β-lactamases (ESBLs) are a class of enzymes that mainly confer resistance to beta-lactam antibiotics, including SHV, TEM, CTX-M and PER. Among them, the CTX-M has been reported to be the predominant type of ESBLs in various Enterobacteriaceae. Since the CTX-M-55 first appeared in India, it has been found in countries worldwide through the transmission of many mobile genetic elements. Recently, considering the increasing detection rate of *bla*_CTX-M-55_ in China, many researches were performed about its characteristics.

Currently, colistin is widely used in clinical practice, mainly for treating infections caused by CRE (Durante-Mangoni et al., 2019). However, the mobile colistin resistance gene *mcr-8* significantly affects the therapeutic efficacy of colistin and the prognosis of patients with associated infections (Phetburom et al., 2021). In 2016, *mcr-8* was first identified in *K. pneumoniae* (Wang et al., 2018). Several *mcr-8* variants have been reported in *K. pneumoniae*, *Klebsiella quasipneumoniae*, *Raoultella ornithinolytica*, and *Enterobacter cloacae,* including *mcr-8.1 mcr*-*8.4* (Wang et al., 2019, 2022; Yang et al., 2019).

The spread of the *mcr* genes into CRE, which has been reported globally, is of great clinical concern, leading to the emergence of true pan-drug-resistant pathogens (Mediavilla et al., 2016; Zheng et al., 2017; Han S. et al., 2020; Chen et al., 2022). Meanwhile, the isolation and culture of such pathogen coharboring *mcr* and carbapenemase-encoding gene from the blood sample is rare. Accordingly, our work aims to describe the antimicrobial susceptibility, plasmid characteristics and genomic features of a *K. pneumoniae* strain co-producing OXA-181, CTX-M-55, and MCR-8 from China for the first time.

## Materials and methods

### Species identification and antimicrobial susceptibility testing

Isolates were collected from a tertiary hospital in Zhengzhou, Henan province, China, during our routine surveillance of CRE. Species identification was performed by matrix-assist laser desorption ionization time-of-flight mass spectrometry (MALDI-TOF/MS) (Bruker Daltonik GmbH, Bremen, Germany). The mobile colistin resistance genes *mcr-1* to *mcr-8* and the major carbapenemase genes, such as *bla*_KPC_, *bla*_NDM_, *bla*_OXA-48_, *bla*_VIM_, and *bla*_IMP_, were identified using PCR, as described previously (Zheng et al., 2019b; Liang et al., 2021).

The susceptibility of *K. pneumoniae* 5589 and its transconjugants to antibiotics was tested using the agar dilution method, except for the polymyxins, which was performed using the broth microdilution method (Liu et al., 2021). The results were interpreted based on the Clinical and Laboratory Standards Institute (CLSI) and the European Committee for Antimicrobial Susceptibility Testing (EUCAST) guidelines. *K. pneumoniae* ATCC700603 and *Escherichia coli* ATCC25922 were used as the quality control.

### Plasmid analysis and conjugation assay

The number and size of plasmids in *K. pneumoniae* 5589 were detected by S1-PFGE of total DNA (Chi et al., 2020). The locations of plasmids harboring the *bla*_OXA-181_ and *mcr-8* were determined by Southern blotting and hybridization with digoxigenin-labeled specific probes. Furthermore, rifampin-resistant *P. aeruginosa* PAO1Ri was used as a recipient bacterium in transformation conjugation experiments to investigate whether the plasmids can transfer (Liu et al., 2021). The transconjugants which showed growth on Mueller-Hinton medium simultaneously containing 300 mg/L rifampicin and 2 mg/L meropenem were identified by MALDI-TOF/MS. The existence of *bla*_OXA-181_ and *mcr-8* in transconjugants was detected by PCR and the antimicrobial susceptibility testing of transconjugants to confirm whether the plasmids carrying target genes were successfully transferred.

### Whole-genome sequencing and analysis

The genome of *K. pneumoniae* 5589 was extracted using a specific bacterial DNA Kit (QIAGEN, Hilden, Germany). To better understand the genetic features, DNA sequencing was performed on the Illumina NovaSeq 6000 (Illumina, San Diego, CA, United States) and the Oxford Nanopore (Oxford Nanopore Technologies, Oxford, United Kingdom) platform (Bao et al., 2022). Then, the whole genome was annotated with Prokka. Additionally, the acquired ARGs were detected by ResFinder 4.1^1^, and the plasmid replicon type was identified by PlasmidFinder 2.1.^2^ The transposon and insertion sequence were detected using the ISFinder database.^3^ Finally, the circular comparison images of multiplex plasmids were generated by BLAST Ring Image Generator (BRIG). The linear comparison figures of multiple genomic loci surrounding the *bla*_OXA-181_ and *mcr-8* were generated by Easyfig 2.0 software (Sullivan et al., 2011).

## Results

### Isolation of *Klebsiella pneumoniae* 5589 and antimicrobial susceptibility testing

Carbapenem-resistant *K. pneumoniae* 5589 was isolated from a blood sample of a patient who was hospitalized for myelodysplastic syndrome (MDS). During his hospitalization, the patient developed thrombocytopenia, high fever and groin infection. Subsequently, the patient’s condition was controlled with a normal body temperature after the biapenem and tigecycline treatment.

The antimicrobial susceptibility profiles of *K. pneumoniae* 5589 and transconjugants were demonstrated in Table 1. *K. pneumoniae* 5589 was resistant to multiple antibiotics such as aztreonam, imipenem, meropenem, ceftriaxone, cefotaxime, ceftazidime, levofloxacin, ciprofloxacin, gentamicin, piperacillin-tazobactam, cefepime and polymyxin B, but remained susceptible to amikacin. Moreover, the transconjugants 5589-PAO1Ri showed a similarity antibiotic resistance profile to *K. pneumoniae* 5589 but was intermediate to imipenem and sensitive to ciprofloxacin and gentamicin.

**Table 1:** MIC values of antimicrobials for *Klebsiella pneumoniae* 5589, transconjugant 5589-PAO1Ri, and recipient strain PAO1Ri.

Antimicrobials	MIC values (mg/L)		
	*K. pneumoniae* 5589	5589-PAO1Ri	PAO1Ri
Aztreonam	>128 (R)	64 (R)	1 (S)
Imipenem	8 (R)	4 (R)	4 (I)
Meropenem	4 (R)	2 (R)	0.25 (S)
Ceftriaxone	>128 (R)	>128 (R)	4 (S)
Cefotaxime	>128 (R)	>128 (R)	8 (S)
Ceftazidime	>128 (R)	>128 (R)	1 (S)
Levofloxacin	>64 (R)	16 (R)	1 (S)
Ciprofloxacin	>64 (R)	1 (S)	1 (S)
Amikacin	2 (S)	2 (S)	2 (S)
Gentamicin	128 (R)	1 (S)	1 (S)
Piperacillin-tazobactam^a^	>128 (R)	8 (R)	1 (S)
Cefepime	32 (R)	4 (R)	1 (S)
Polymyxin B	8 (R)	4 (R)	1 (S)

a Tazobactam at a fixed concentration of 4 mg/L.

### Genomics features of *Klebsiella pneumoniae* 5589

The *K. pneumoniae* 5589 genome contains a 5,279,178 bp circular chromosome with an average GC content of 57.5% and three plasmids of different sizes from 51,479 bp to 290,720 bp (Supplementary Table S1). WGS revealed that *K. pneumoniae* 5589 was identified as ST273, which belongs to the clonal group 147. By researching the ARGs on ResFinder, 48 acquired resistance genes were detected (Supplementary Table S1). The chromosome of strain *K. pneumoniae* 5589 was found to harbor ARGs which confer resistance to beta-lactams (*bla*_SHV-11_, *bla*_SHV-67_), fosfomycin (*fosA*), chloramphenicol (*OqxA*, *OqxB*). Moreover, it carried multiple virulence genes such as coding for outer membrane receptor (*fepA*), transcriptional regulator (*fimK*), regulator protein (*ykgK*) and transcriptional activator (*mrkH*).

### Characterization of plasmid bearing *bla*_OXA-181_

**Figure 1:**
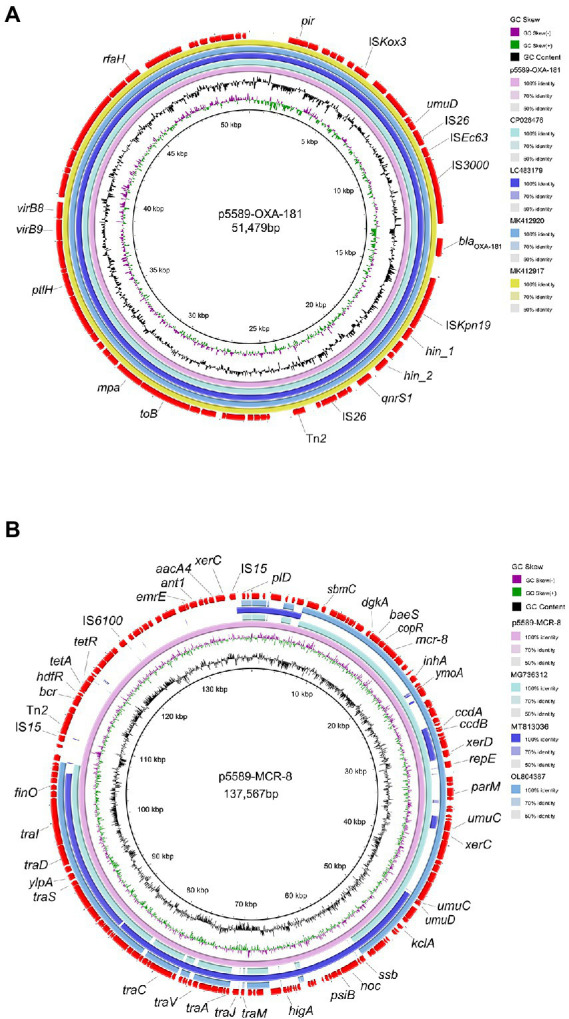
Comparative analysis of plasmids p5589-OXA-181 and p5589-mcr-8 detected in *Klebsiella pneumoniae* 5589. **(A)** Comparison of *bla*_OXA-181_ bearing plasmid p5589-OXA-181 with pKBN10P04869C (GenBank accession no. CP026476), pNIPH17_0036_1 (GenBank accession no. LC483179), pBC947-OXA-181(GenBank accession no. MK412920) and pABC264-OXA-181(GenBank accession no. MK412917). **(B)** Comparison of mcr-8-carrying plasmid p5589-MCR-8 with pKP91(GenBank accession no. MG736312), pKQBSI104-1(GenBank accession no. MT813036) and pKP3(GenBank accession no. OL804387).

S1-PFGE and southern blot results revealed that the resistance gene *bla*_OXA-181_ was located on a 51,479 kb plasmid (p5589-OXA-181), which belongs to IncX3-ColKP3 with a GC content of 46% (Supplementary Figure S1). The plasmid carrying *bla*_OXA − 181_ was successfully transferred to a *P. aeruginosa* PAO1Ri recipient strain. The plasmid p5589-OXA-181 carries not only the *bla*_OXA-181_ but also *qnrS1*, which enables the strain to be resistant to ciprofloxacin. According to the result of the BLAST search, plasmid p5589-OXA-181 was almost identical to pNIPH17_0036_1 (accession no: LC483179), pKBN10P04869C (accession no: CP026476), pABC264-OXA-181 (accession no: MK412917) and pBC947-OXA-181 (accession no: MK412920) with the similarity between 99% and 100% (Figure 1A). The genetic environment analysis showed *bla*_OXA-181_ was located on a composite transposon surrounded by two copies of insertion sequence IS*26*. A similar region can be seen in *E. coli* plasmid pKBN10P04869C (accession no: CP026476), and *E. coli* plasmid pABC264-OXA-181 (accession no: MK412917). In plasmid p5589-OXA-181, the gene *repA1* is responsible for encoding ColKP3-type replication initiation protein, and the IS*Kpn19* fragment is located downstream of *bla*_OXA-181_, while IS*3000* is located on the upstream (Supplementary Figure S2A). Many other functional genes such as encoding DNA topoisomerase (*topB*), proteasome-associated ATPase (*mpa*), type IV secretion system protein (*ptlH*, *virB9*, *virB8*), transcription antitermination protein (*rfaH*) are distributed on the backbone.

### Characterization of plasmid bearing *mcr-8*

The *mcr-8* gene was carried by another plasmid of size 137,567 kb (p5589-mcr-8) with the replicon type of IncFIA-FII (Supplementary Figure S1). Meanwhile, we obtained the transconjugant harboring *mcr-8* successfully. The plasmid p5589-mcr-8 contained additional genes that make strain exhibit resistance to multiple antibiotics, such as bleomycin (*bleO*), spectinomycin (*aadA16*), ciprofloxacin (*aac (6′)-Ib-cr*, *qnrB91*), rifampicin (*arr-3*), azithromycin (*mph(A)*), trimethoprim (*dfrA27*), sulfamethoxazole (*sul1*), tetracycline (*tet(A)*), chlorhexidine (*qacE*), chloramphenicol (*floR*). On the other hand, the plasmid p5589_MCR-8 showed great similarity to pKP91(65%coverage and 99.70%identity; accession no: MG736312), pKQBSI104-1(52%coverage and 99.47%identity; accession no: MT813036), pKP3(65%coverage and 99.86%identity; accession no: OL804387; Figure 1B).

The inspection of the genetic regions revealed that *mcr-8* in this work was similar to that of the *mcr-8.1* gene in plasmid pK91 and pKP3. The upstream was the IS*903B* and other functional genes (*inhA*, *YmoA*). At the same time, the downstream of *mcr-8* were the transfer or transcription-associated genes (*copR*, *sasA dgkA*) and encoding β-lactamase gene (*ampc*) (Supplementary Figure S2B). The p5589-mcr-8 backbone carried regions responsible for toxin-antitoxin (TA) systems (*ccdA*, *ccdB*, *higA*, *ylpA*), replication (*repE*), mobilization (*tra*, *xerD*, *xerC*, *klcA*, *finO*) and stability (*parM*, *umuC*, *umuD*, *ssb*, *noc*, *psiB*). Other genes encode enzymes associated with DNA replication (*sbmC*, *ant1*, *aacA4*, *pld*), and proteins associated with the resistance and transport of multidrug (*bcr*, *tetA*, *tetR*, *emrE*) also can be found.

## Discussion

OXA-181-producing Enterobacteriaceae have been reported in several countries, including Portugal, South Africa, and Singapore, but have rarely been described in China, where *Klebsiella pneumoniae* carbapenemase (KPC) is the major carbapenemase (Han R. et al., 2020; Chew et al., 2021). To our knowledge, OXA-181 has not emerged in China until 2015, and the report of *bla*_OXA-181_ in China is still uncommon (Qin et al., 2018). Infections caused by OXA-181 in nonendemic areas were often associated with the travelling of patients to endemic areas (Chudejova et al., 2021). However, the identification of *bla*_OXA-181_ in this work was from a patient without a history of foreign residence, which indicated its wider dissemination than previously anticipated.

So far, several studies have reported plasmids carrying *bla*_OXA-181_ with different replicons, such as IncX3, IncA/C, ColE, IncT, IncN, IncFIIK and ColKP3 (Villa et al., 2013; Naha et al., 2021). But the most common plasmid in China is the IncX3 type which has a similar genetic environment to others (Liu et al., 2020). The similar *bla*_OXA-181_ bearing IncX3 plasmid further highlights the role of IncX3-type plasmid as an irreplaceable vector of ARGs (Santos Tufic-Garutti et al., 2022). In p5589-OXA-181, *bla*_OXA-181_ was found on a composite transposon which was considered to facilitate its horizontal transmission (Supplementary Figure S2A). Therefore, the absence of upstream mobile element IS*Ecp1* was detected in p5589-OXA-181, which was consistent with other studies (Naha et al., 2021). Generally, IS*Ecp1* plays an essential role in the transmission of ARGs; its absence may affect transposase activity and the maintenance of resistance genes on a plasmid (Potron et al., 2013; Naha et al., 2021).

The colistin resistance mechanism in Enterobacteriaceae is complicated and has not been wholly investigated (El-Sayed Ahmed et al., 2020). In general, the resistance to colistin can be acquired by intrinsic mutation or adaptation mechanisms and the horizontal transfer of *mcr* gene and its variants (Moffatt et al., 2019). Since the initial report of *mcr-8*, this gene has been discovered in Enterobacteriaceae isolates from humans, animals and various environments worldwide (Anyanwu et al., 2020; Ngbede et al., 2020).

There have already been several studies about the genetic context analysis of *mcr-8* (Wu et al., 2020). The *mcr-8* gene was firstly recognized on a typical IncFII-type plasmid pKP91, with a conservative region flanked by IS*903B*, but in p5589-mcr-8, the IS*903B* located on the upstream was absent (Supplementary Figure S2B; Wang et al., 2018). The *mcr-8*-carrying plasmid in *Raoultella ornithinolytica* also harbored only one copy of IS*903B* located upstream (Wang et al., 2019). In addition, Farzana has reported the upstream IS*903B* of *mcr-8* in ST15 *K. pneumoniae* was replaced by IS*Kpn21* (Farzana et al., 2020). Thus, it was reasonable to speculate that the upstream IS*903B* surrounding *mcr-8* is unstable and replaceable. However, additional studies focusing on this are warranted.

*K. pneumoniae* 5589 in this work also carried *bla*_CTX-M-55_, which was frequently found in *E. coli* (Zhang et al., 2014; Birgy et al., 2018; Feng et al., 2019). Hence, we could also pay more attention on *bla*_CTX − M − 55_-positive *K. pneumoniae* to better understand the molecular epidemiology of CTX-M-55 in China. Simultaneously, *K. pneumoniae* 5589 was assigned to ST273, which was recognized as the reservoir of many carbapenemase genes, including *bla*_KPC_, *bla*_VIM_, *bla*_NDM_ and *bla*_IMP_ (Chou et al., 2016; Liu et al., 2018). ST273 was divided into the specific clonal group 147, which had a high epidemic potential (Rodrigues et al., 2022). The original detection of ST273 was in Europe and had been gradually identified in Italy, Norway, and Russia, even causing the outbreak in Southeast Asia. Furthermore, whether the ST273 could influence the epidemiology of *bla*_OXA-181_ and *mcr-8* remains unknown, and necessary attention should be paid to these sequence types to avoid epidemic outbreaks.

Previous studies have reported the co-producing of OXA-181 and other carbapenemases, such as co-harboring *bla*_OXA-181_ and *bla*_NDM-5_ in Nepal, *bla*_OXA-181_ and *bla*_NDM-1_ in French, and *bla*_OXA-181_ and *bla*_KPC-121_ in Italy (Sherchan et al., 2020; Gaibani et al., 2022). Remarkably, the spread of *mcr* gene into CRE resulting the accumulation of multidrug resistance genes. Nevertheless, the study about the co-carriage of *bla*_OXA-181_ and *mcr-8* is limited, except for a report of an *Escherichia coli* co-producing OXA-181 and MCR-1 (Pulss et al., 2017). Identifying the isolate co-carrying *bla*_OXA-181_ and *mcr-8* in *K. pneumoniae* reminds us that persistence detection and further exploration are needed to prevent the emergence and evolution of such MDR isolates.

## Conclusion

In summary, our work firstly described the co-occurrence of OXA-181, CTX-M-55, and MCR-8 in *K. pneumoniae*. Our study also characterized the *bla*_OXA-181_ and *mcr-8*-carrying plasmids, which contribute to exploring the transmission mechanism. The appearance of such clinical isolates producing carbapenemases and MCR narrows the therapeutic options and reveals the severe situation of antimicrobial resistance. Continuous observation and exploration are essential to control its spread.

## Data availability statement

The datasets presented in this study can be found in online repositories. The names of the repository/repositories and accession number(s) can be found in the article/Supplementary material.

## Author contributions

XG and BZ conceived and designed the experiments. HG, JQ, RC, CL, and JZ collected samples and performed the experiments. HX, RL, and XH analyzed the data. HG wrote the manuscript. BZ reviewed and finalized the manuscript. All authors contributed to the article and approved the submitted version.

## Funding

This work was supported by research grants from Henan Science and Technology Department (192102310059), the National Natural Science Foundation of China (82072314), the Research Project of Jinan Microecological Biomedicine Shandong Laboratory (JNL-2022011B), the Fundamental Research Funds for the Central Universities (2022ZFJH003), CAMS Innovation Fund for Medical Sciences (2019-I2M-5-045), Henan Province Medical Science and Technology Research Project Joint Construction Project (LHGJ20190232), and Zhejiang Provincial Natural Science Foundation of China (LQ20H200003).

## Conflict of interest

The authors declare that the research was conducted in the absence of any commercial or financial relationships that could be construed as a potential conflict of interest.

## Publisher’s note

All claims expressed in this article are solely those of the authors and do not necessarily represent those of their affiliated organizations, or those of the publisher, the editors and the reviewers. Any product that may be evaluated in this article, or claim that may be made by its manufacturer, is not guaranteed or endorsed by the publisher.

## Footnotes

^1^ https://cge.food.dtu.dk/services/ResFinder/

^2^ https://cge.food.dtu.dk/services/PlasmidFinder/

^3^ http://www-is.biotoul.fr/
