# Supplementary material for: First report of Klebsiella pneumoniae co-producing OXA-181, CTX-M-55, and MCR-8 isolated from the patient with bacteremia
Source: Front Microbiol. 2022 Oct 14;13:1020500. doi: 10.3389/fmicb.2022.1020500 (PMC9614159; doi:10.3389/fmicb.2022.1020500)

Supplementary Material

**Supplementary Table S1.** Features of chromosome and plasmids harbored by *K. pneumoniae* 5589.

| **Table S1**. Features of chromosome and plasmids harbored by *K. pneumoniae* 5589. | | | | | |
| --- | --- | --- | --- | --- | --- |
| Sample name | Size (bp) | ST | Inc group | Antimicrobial resistance genes | GenBank accession no. |
| chromosome | 5279,178 | 273 | NA^a^ | *bla*_SHV-11_, *bla*_SHV-67_, *fosA*, *OqxA*, *OqxB* | CP102077 |
| p5589-CTX-M-55 | 290,720 | NA | IncHI1B-IncR | *bla*_CTX-M-55_, *bla*_TEM-1B_, *bla*_TEM-141_, *bla*_TEM-206_, *bla*_TEM-214_, *qacL*, *msr(E)*, *mph(E)*, *fosA3*, *cmlA1*, *sul3*, *aph(6)-Id*, *aadA2b*,*aph(3'')-Ib*, *aph(4)-Ia*, *aadA1*, *aac(3)-IV* | CP102078 |
| p5589-MCR-8 | 137,567 | NA | IncFIA-FII | *mcr-8*, *qacE*, *ARR-3*, *mph(A)*, *dfrA27*, *floR*, *sul1*, *aac(6')-Ib-cr*, *aadA16*, *bleO*, *qnrB91*, *tet(A)* | CP102079 |
| p5589-OXA-181 | 51,479 | NA | IncX3-ColKP3 | *bla*_OXA-181_, *qnrS1* | CP102080 |

^a^ NA, not applicable.

**Supplementary Figure S1** Plasmid profiles of *K. pneumoniae* 5589. **(A)** Plasmid size determination by S1-PFGE, with Salmonella enterica serotype Braenderup H9812 as the size marker. **(B)** Southern blotting hybridization with an mcr-8-specific probe. **(C)** Southern blotting hybridization with a blaOXA-181-specific probe.


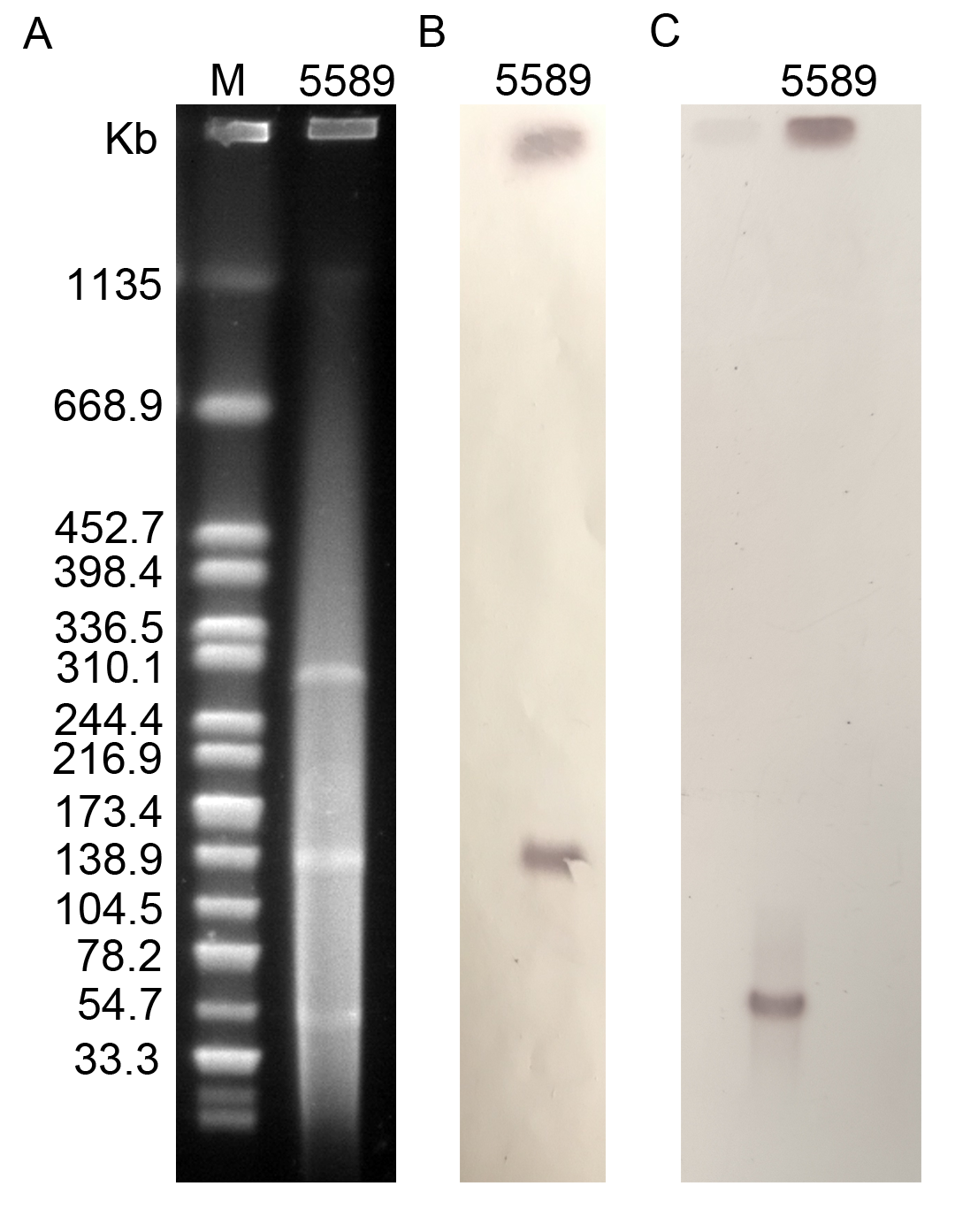


**Supplementary Figure S2** The genetic context of blaOXA-181 and mcr-8 gene. (A) Comparison of genes surrounding blaOXA-181 on p5589-OXA-181, pKBN10P04869C (GenBank: CP026476) and pABC264-OXA-181(GenBank: MK412917). (B) Comparison of genes surrounding mcr-8 on p5589-mcr-8, pKP91(GenBank accession no. MG736312), pKP3(GenBank accession no. OL804387). Open reading frames (ORFs) are shown as arrows and indicated according to their putative functions. Pink indicates antimicrobial resistance genes, yellow indicates genes related to mobile elements, and the light green represents other functional genes. The hypothetical protein encoded genes are colored grey. Regions with a high degree of homology are indicated by purple shading.


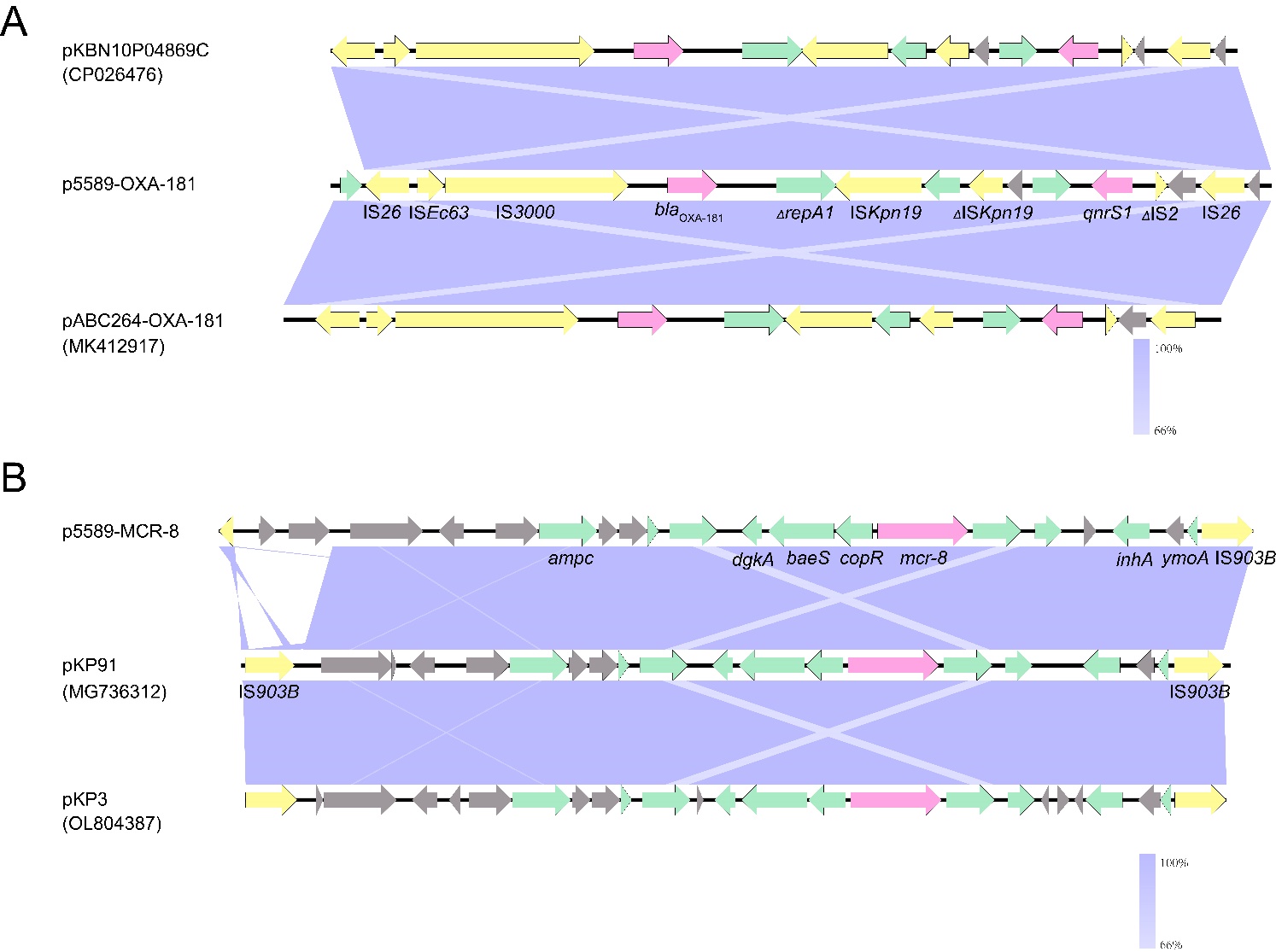

Supplement: Supplementary file 1 [file Data_Sheet_1.docx]
